# Psychiatric Morbidities of Elderly Out-patients Attending Various Outreach Clinics in Gandaki Province of Nepal: A Descriptive Cross-sectional Study

**DOI:** 10.31729/jnma.4996

**Published:** 2020-05

**Authors:** Jai Bahadur Khattri, Srijana Thapa Godar, Anil Subedi, Shweta Tirkey

**Affiliations:** 1 Department of Psychiatry, Manipal College of Medical Sciences, Pokhara, Nepal; 2 Department of Ophthalmology, Manipal College of Medical Sciences, Pokhara, Nepal

**Keywords:** *aging*, *cross-sectional studies*, *Nepal*, *prevalence*

## Abstract

**Introduction:**

Dramatic growth of the aging population resulted in an increased number of mental disorders and such data are limited in Nepal. The objective is to find out the psychiatric morbidities of elderly out-patients attending Baglung, Kusma, Walling, and Dumre out-reach clinics in Gandaki Province of Nepal.

**Methods:**

This was a descriptive cross-sectional study conducted in 392 patients attending out-reach clinics of Baglung, Kusma, Walling, and Dumre of Gandaki Province for one year with the convenient sampling method. Ethical approval was taken from the Institutional Review Committee of Manipal College of Medical Sciences, Pokhara. The diagnosis was done according to the International Classification of Disease-10 guidelines. Epi-info 7 was used and point estimate at 95% Confidence Interval was calculated along with frequency and proportion for binary data and the analysis was done.

**Results:**

The prevalence of neurotic, stress-related, and somatoform disorders 145 (37.0%) was maximum followed by mood disorders 111 (28.3%). Maximum cases were in between 66 and 75 years, Brahmin and Chhetri caste, females, married, and from an extended nuclear family. There is more prevalence of female gender in all psychiatric diagnoses except in mental and behavioral disorders due to psychoactive substance use. There is more prevalence of age groups of patients less than or equal to 75 years in all the psychiatric diagnosis.

**Conclusions:**

Neurotic, stress-related, and somatoform disorders were the most common diagnosis. The risk of development of certain disorders based on gender and age group of the patients would be helpful for case identification.

## INTRODUCTION

The aging of the populations has become a worldwide phenomenon.^1^ This phenomenon has extensive consequences.^2^ Population aging is producing changes to the demographics of the developing countries like Nepal.^3^ The people aged above 65 years in Nepal comprises 5.42% in 2011 which is projected to increase to 6.79% of the total population in 2031.^4^

This phenomenon has dramatic consequences for public health, health care financing, and delivery systems in the whole world. Mental disorders are common chronic conditions among elderly people, and the absolute number of subjects with psychiatric disorders will increase dramatically soon worldwide.^5^

A plan is needed for provisions of services to elderly persons with mental disorders.^6^ The objective is to find out the psychiatric morbidities of elderly out-patients attending Baglung, Kusma, Walling, and Dumre outreach clinics of the Gandaki Province of Nepal.

## METHODS

This descriptive cross-sectional study was conducted in all the elderly patients of above 65 years who attended the psychiatry out-patients department (OPD) of outreach clinics of Baglung, Kusma, Walling, and Dumre in the Gandaki Province of Nepal for one year (from November 2018 to October 2019). An equal number of patients (n= 98) were selected from each of the four clinics. All the elderly patients of above 65 years who attended Psychiatry OPD of out-reach clinics of Baglung, Kusma, Walling, and Dumre in the Gandaki Province of Nepal were included in the study. The elderly patients with acute medical or surgical emergency conditions were excluded from the study. The ethical approval of the study was taken from the Institutional Review Committee of Manipal College of Medical Sciences, Pokhara (MEMG/IRC/192/GA) before the start of the study.

The sample size was calculated by using the following formula,

n= Z^2^ × p × q/e^2^

= (1.96)^2^ × (0.375 × 0.625)/ (0.05)^2^

= 360

Where,

n= sample size
Z= 1.96 at 95% CI
p= prevalence, 37.5%^7^
q= 1-p
e= margin of error, 5%

By adding 32 more samples (9% as non-response rate), the final sample size was calculated to be 392.

The written informed consent was taken from the patients. The consent was taken from the relatives of the patients in case if the patient was unable to give consent because of an unstable mental state. The confidentiality of the participants was maintained.

The convenience sampling method was used. Selection and information bias has been minimized as possible. Data entry and analysis was done in Epi-info version 7. Point estimate at 95% confidence interval was calculated along with frequency and proportion for binary data and analysis was done.

A self-designed proforma was used to record the socio-demographic profile of the patients. The diagnosis of the elderly patients was based on International Classification of Disease-10 guidelines.^8^

## RESULTS

A total of 392 consecutive elderly patients was selected for the study. The prevalence of neurotic, stress-related, and somatoform disorders 145 (37.0%) was maximum followed by mood disorders 111 (28.3%).

The sociodemographic profile of the patients is shown (Table 1).

**Table 1:** Sociodemographic profile of the patients.

Socio-demographic variables	n (%)
**Age groups (in years)**	
66-75	336 (85.7)
76-85	51 (13.0)
>86	5 (1.3)
**Gender**	
Female	214 (54.6)
Male	178 (45.4)
**Caste**	
Brahmin and Chhetri	239 (61.0)
Mongolian	93 (23.7)
Newar	13 (3.3)
Others	47 (12.0)
**Marital status**	
Married	249 (63.5)
Separated	23 (5.9)
Unmarried	8 (2.0)
Widow/widower	112 (28.6)
**Type of family**	
Extended nuclear	219 (55.9)
Joint	133 (33.9)
Nuclear	40 (10.2)
Total	392 (100)

Mean age±SD= 69.86±5.04 years

The majority of the patients were from the age group between 66 and 75 years 336 (85.7%), females 214 (54.6%), Brahmin & Chhetri caste 239 (61.0%), married 249 (63.5%) and from the extended nuclear family 219 (55.9%). The mean age of the patient was 69.86±5.04 years.

The distribution of respondents as per the diagnosis based on International Classification of Diseases-10 guidelines is shown (Table 2). The majority of the cases were found to be suffering from neurotic, stress-related, and somatoform disorders 145 (37.0%). This was followed by a mood disorder 111 (28.3%).

**Table 2:** Diagnosis according to ICD-10 guidelines of the patients.

ICD-10 diagnosis	ICD-10 code	n (%)
Organic, including symptomatic, mental disorders	F00-F09	30 (7.7)
Mental and behavioral disorders due to psychoactive substance use	F10-F19	15 (3.8)
Schizophrenia, schizotypal and delusional disorders	F20-F29	12 (3.1)
Mood disorders	F30-F39	111 (28.3)
Neurotic, stress-related and somatoform disorders	F40-F48	145 (37.0)
Headache	G43&44	3 (0.8)
Seizure disorder	G40&41	4 (1.0)
Movement disorders	G25	6 (1.5)
Other organic disorders		62 (15.8)
No diagnosis		4 (1.0)
Total		392 (100.0)

The distribution of the psychiatric diagnosis according to the gender of the patients is shown (Table 3). All the psychiatric diagnosis was prevalent among female gender except in mental and behavioral disorders due to psychoactive substance use.

**Table 3:** Distribution of the diagnosis according to the gender.

Diagnosis	Gender		
	Female n(%)	Male n(%)	n(%)
Organic, including symptomatic, mental disorders	17 (56.66)	13 (43.33)	30 (100)
Mental and behavioral disorders due to psychoactive substance use	3 (20.00)	12 (80.00)	15 (100)
Schizophrenia, schizotypal and delusional disorders	8 (66.66)	4 (33.33)	12 (100)
Mood disorders	63 (56.75)	48 (43.24)	111 (100)
Neurotic, stressrelated and somatoform disorders	93 (64.13)	52 (35.86)	145 (100)

The distribution of the psychiatric diagnosis as per the age groups is shown (Table 4). All psychiatric illness is prevalent among patients less than or equal to 75 years.

**Table 4:** Distribution of the diagnosis according to the age groups.

Diagnosis	Age groups (years)		
	<75 n (%)	>75 n (%)	Total n (%)
Organic, including symptomatic, mental disorders	18 (60.00)	12 (40.00)	30 (100)
Mental and behavioral disorders due to psychoactive substance use	14 (93.33)	1 (6.66)	15 (100)
Schizophrenia, schizotypal and delusional disorders	12 (100)	0	12 (100)
Mood disorders	102 (91.89)	9 (8.10)	111 (100)
Neurotic, stressrelated and somatoform disorders	130 (89.65)	15 (10.35)	145 (100)

## DISCUSSION

The elderly population is rapidly growing as compared to other populations around the world in the present century. The health sectors of the countries like Nepal should have proper planning to deal with the growing medical and psychiatric needs of this population to reduce the sudden negative impact of this population in the health infrastructures in the future.

There were 392 cases aged more than 65 years who visited the out-reach clinics are selected for the study. The maximum number of patients falls in the age group between 66 and 75 years 336 (85.7%). Different studies also found maximum cases in the younger elderly age-group.^9-19^ The mean age of the patients was 69.86 years with a standard deviation of 5.04 years in the current study. This finding was also following a study conducted in Nepal.^17^,^20^ This could be explained by the life expectancy of the Nepalese population and less number of patients is likely to survive in the advance age.^4^

There are more females as compared to males in the current study. This finding was following the studies done both in Nepal and India.^15^,^16^,^19^,^21^ However, the different studies found more male respondents in their sample of the population 10-12,14,17,20

Majority of the respondents were Brahmins & Chhetri 239 (61.0%) followed by Mongolians 93 (23.7%), others (Dalits) 47 (12.0%) and Newars 13 (3.3%) in the current study. A similar trend has been found in the different studies conducted in Nepal.^14^,^16^,^17^,^20^ According to one study, the Brahmins and Chhetri are more aware of psychiatric illness and their treatment due to the privilege of higher education/ socio-economic status.^20^ There is a higher proportion of the distribution of Brahmin and Chhetri caste in the total national census of Nepal as well.^4^

Maximum cases in the out-reach clinics were married in the present study. The different studies also found the maximum number from the married group.^9^,^15^,^17^,^19^,^20^ Better support system of the married patients can explain this finding.^20^ However, a well-designed population-based case-control study is needed to establish this finding.

In the present study, about 55.9% and 33.9% of the cases come from extended nuclear and joint family respectively. This finding also consistent with the one study conducted in Nepal.^17^ The study conducted in India concluded that family setting continues to be available for the elderly in the developing country.^9^

This study found that the maximum proportions of elderly citizens are suffering from neurotic, stress-related, and somatoform disorders (37.0%). One study was done in a rural setting inside Nepal also found maximum respondents to be suffering from somatization and generalized anxiety.^22^ However, the different studies found maximum senior citizens to be suffering from mood disorders.^13^,^15^,^16^,^18^,^23^ One study conducted in Nepal found maximum patients to be suffering from psychosis.^14^ These overall findings reflect that if the study is conducted in the rural area^22^ or close to the rural area (like in this study), there is a high prevalence of neurotic disorders. This is just the possible hypothesis which needs to confirm/reject with large scale comparative studies. This could be due to the fact that long standing somatic symptoms of neurotic disorders make the patient to visit multiple hospitals to relieve their distress immediately.^24^

The prevalence of organic, including symptomatic, mental disorders was 7.7% in the present study. The different studies found prevalence of 11.9%,^18^12%,^16^ 15%,^17^,^23^and 15.9%.^13^

The diagnosis of mental and behavioral disorders due to psychoactive substance use constitutes 3.8% of the total sample in the current sample. This patient was diagnosed to be suffering from alcohol dependence syndrome. Other studies done previously in Nepal found prevalence of 5%,^25^10.5%,^14^12.9%,^15^14%,^23^18.1%^13^ and 19%.^16^

The prevalence of schizophrenia, schizotypal, and delusional disorders was 3.1% in the current sample. The prevalence of schizophrenia ranges from 6% to 33.3% according to different studies have done both inside Nepal and India.^13-16^,^18^,^23^

The prevalence of mood disorder (which includes both depressive episodes and bipolar disorders) in the current study was 28.3%. The prevalence found in other studies were 19.5%,^14^ 32.5%,^23^ 39.1,^13^ 44.0%,^18^ 44.8%^15^ and 46%.^16^ The study conducted in tertiary care hospital in Nepal found a prevalence of depression of 53.2% according to the Geriatric Depression Scale.^26^

The proportion of elderly patients to be suffering from neurotic, stress-related, and somatoform disorders was 37.0% in this study. The other study done in Nepal found a prevalence of 23.3% and 2.5% of anxiety disorder and somatoform disorder respectively.^23^ Two studies done in the Eastern region of Nepal found a prevalence of 22%^16^ and 13.8%.^15^

In the gender distribution of the current study samples, the psychiatric diagnosis was more prevalent in females as compared to males except in mental and behavioral disorders due to psychoactive substance use. One study conducted in the same Province area as the current study also found more elderly males to be suffering from alcohol dependence syndrome.^25^ One study done in Nepal found more prevalence of alcohol dependence and dementia in the male population.^13^ There is more prevalence of all psychiatric diagnoses in the patients less than or equal to 75 in this study. One study found that except for dementia, there is more prevalence of psychiatric diagnosis at the age of less than or equal to 75 years as compared to above 75 years.^23^ This overall finding might just be due to the small sample size of patients above 75 years in the current study.

The reasons for the study findings of the different studies might be due to the difference in the sample characteristics of the study populations. The different study design and confounding factors in various studies may lead to different findings. The use of different assessment tools and study settings (out-patient, inpatient, and rural setting) might be the other reason for the difference in the study findings.

There are limitations to this study. The use of ICD for diagnosis of psychiatric disorder in an elderly sample may underestimate its true prevalence. Also, as this study was conducted in the outpatient department settings, the finding cannot be generalized to the entire population.

## CONCLUSIONS

The study found that the majority of the elderly patients who visited the out-reach clinics were from the age groups between 66 and 75 years, Brahmin and Chhetri caste, females, married, and living in the extended nuclear family. The maximum patients were also found to be suffering from the neurotic, stress-related, and somatoform disorder which is followed by a mood disorder. This study finding would be helpful in the identification and holistic management of elderly patients. Further large scale, case-control, and analytical design studies in the community setting in a different region of Nepal are required before generalizing the result of this study.

## Conflicts of Interest:

**None.**
